# Risk factors for multidrug-resistant pathogens in bronchiectasis exacerbations

**DOI:** 10.1186/s12879-017-2754-5

**Published:** 2017-09-30

**Authors:** Rosario Menéndez, Raúl Méndez, Eva Polverino, Edmundo Rosales-Mayor, Isabel Amara-Elori, Soledad Reyes, José Miguel Sahuquillo-Arce, Laia Fernández-Barat, Victoria Alcaraz, Antoni Torres

**Affiliations:** 1 Pneumology Department, Hospital Universitario y Politécnico La Fe / Instituto de Investigación Sanitaria (IIS) La Fe, Universidad de Valencia, Valencia, Spain; 2 Fundació Clínic, Institut D’ Investigacions Biomèdiques Agustí Pi i Sunyer (IDIBAPS), Barcelona, Spain; 3 Microbiology Department, Hospital Universitario y Politécnico La Fe / Instituto de Investigación Sanitaria (IIS) La Fe, Valencia, Spain; 4 Pneumology Department, Hospital Clínico y Provincial, Universidad de Barcelona, Institut D’ Investigacions Biomèdiques Agustí Pi i Sunyer (IDIBAPS), Barcelona, Spain; 5 Centro de Investigación Biomédica En Red-Enfermedades Respiratorias (CIBERES, CB06/06/0028), Madrid, Spain

**Keywords:** Multidrug-resistant, Pseudomonas, Hospitalization

## Abstract

**Background:**

Non-cystic fibrosis bronchiectasis is a chronic structural lung condition that courses with recurrent infectious exacerbations that lead to frequent antibiotic treatment making this population more susceptible to acquire pathogens with antibiotic resistance. We aimed to investigate risk factors associated with isolation of multidrug-resistant pathogens in bronchiectasis exacerbations.

**Methods:**

A prospective observational study was conducted in two tertiary-care hospitals, enrolling patients when first exacerbation appeared. Multidrug-resistance was determined according to European Centre of Diseases Prevention and Control classification.

**Results:**

Two hundred thirty three exacerbations were included and microorganisms were isolated in 159 episodes. Multidrug-resistant pathogens were found in 20.1% episodes: *Pseudomonas aeruginosa* (48.5%), *methicillin-resistant Staphylococcus aureus* (18.2%) and *Extended spectrum betalactamase + Enterobacteriaceae* (6.1%), and they were more frequent in exacerbations requiring hospitalization (24.5% vs. 10.2%, *p*: 0.016). Three independent multidrug-resistant risk factors were found: chronic renal disease (Odds ratio (OR), 7.60, 95% CI 1.92–30.09), hospitalization in the previous year (OR, 3.88 95% CI 1.37–11.02) and prior multidrug-resistant isolation (OR, 5.58, 95% CI 2.02–15.46). The proportion of multidrug-resistant in the 233 exacerbations was as follows: 3.9% in patients without risk factors, 12.6% in those with 1 factor and 53.6% if ≥2 risk factors.

**Conclusions:**

Hospitalization in the previous year, chronic renal disease, and prior multidrug-resistant isolation are risk factors for identification multidrug-resistant pathogens in exacerbations. This information may assist clinicians in choosing empirical antibiotics in daily clinical practice.

## Background

Multidrug-resistant (MDR) pathogens are a worldwide health threat with clinical negative consequences if inadequately recognized and treated. Non-cystic fibrosis bronchiectasis (BE) is a chronic structural lung condition that facilitates chronic colonization by microorganisms and courses with frequent exacerbations and recurrent infections. [1, 2] This means that patients receive numerous courses with broad-spectrum antibiotics, making them more likely to acquire MDR pathogens.

The main pathogens involved in chronic colonization and acute exacerbations are *Haemophilus influenzae, Streptococcus pneumoniae, Pseudomonas aeruginosa*, and, to a lesser extent, *Enterobacteriaceae*. The incidence and spread of MDR microorganisms among BE patients is worrisome because the antibiotic arsenal is scarce and the most threatening potential MDR pathogens in respiratory patients include *P. aeruginosa* and extended-spectrum betalactamase (ESBL) *Enterobacteriaceae.* These pathogens are difficult to treat because they require different antibiotic regimens to those usually recommended in guidelines.

To our knowledge, no prospective studies have been aimed at identifying independent risk factors for MDR pathogens in BE exacerbations. [3, 4] We hypothesized that MDR exacerbations depend on patient characteristics, including usual treatments and prior health contacts, and that knowledge of these factors may be useful for reducing inappropriate antibiotic treatment.

The aim of our study was to investigate risk factors associated with isolation of multi-drug resistant microorganisms in bronchiectasis exacerbations and their clinical impact on outcome.

## Methods

We conducted a prospective and observational study of adult patients with bronchiectasis attended in the specialized clinic of two tertiary care university hospitals during the period 2011–2015. In our specific specialized clinic, patients are referred from primary care, other hospitals, other specialties or any other medical facilities. We confirmed the diagnosis of bronchiectasis by computerized tomography scan of lungs along with compatible symptoms and aetiology of bronchiectasis had been investigated according to Spanish guidelines [5] previous to study recruitment. Local committees approved the study and patients gave written informed consent (Biomedical research ethics committee Hospital La Fe 2011/0342).

Patients were enrolled in the study when they presented the first exacerbation (after signing the informed consent) and required new antibiotic treatment or hospital admission and no subsequent exacerbations for every patient were included. Exclusion criteria were: a) severe immunosuppression, such as in solid-organ or bone-marrow transplantation or HIV/AIDS, or receiving chemotherapy or other immunosuppressive drugs (≥20 mg prednisone-equivalent per day for 2 weeks or more); b) active tuberculosis; c) cystic fibrosis (CF); d) pulmonary interstitial disease and e) hospitalization in the preceding 21 days.

### Study protocol

Data collected were demographic, diagnosis of BE, smoking, alcohol abuse and flu vaccine status. Comorbidities were also recorded (diabetes, COPD, asthma, heart disease, prior tuberculosis, renal, liver and cerebrovascular diseases) and age-adjusted Charlson score. [6] Data related to prior microorganisms isolation, number of exacerbations in the previous year, bronchiectasis severity scores (BSI, FACED) [7, 8] were also recorded*.* Chronic and concomitant medication included bronchodilators, corticosteroids, theophylline, inhaled/nebulized antibiotics, proton pump inhibitors, long-term oxygen therapy and mucolytic drugs. A history of prior exacerbations and hospitalization during the previous year were also detailed.

### Exacerbation definition and follow-up

The definition of exacerbation according to Spanish guidelines [5] was as follows: acute change in sputum characteristics (increased volume, change of viscosity, purulence) with or without increased dyspnea after ruling out any other causes along with the requirement of a new antibiotic treatment prescribed in our specific clinic and / or unscheduled admission to hospital. We included also exacerbations with new chest x-ray infiltrates diagnosed as pneumonia. The attending physician made the decision to admit to hospital. During the exacerbation episode, data collected were change of initial antibiotic, complications, invasive and noninvasive mechanical ventilation, and mortality. Length of hospital stay was recorded in hospitalized patients and new exacerbations at 1 year of follow-up. Inappropriate antibiotic treatment was considered when pathogens were not susceptible to the prescribed antibiotic with respect to in vitro susceptibility testing. Patients were followed up for visits in the specialized clinic at 30 days, 90 days and 1 year after discharge.

### Microbiological evaluation and diagnosis

The microbiological diagnosis was performed with the following tests: sputum (208 patients), urine antigen test for *S. pneumoniae* (126) and *L. pneumophila* (128), two blood samples (87) and nasopharyngeal swabs (125) (for influenza A and B, parainfluenzae, syncytial respiratory virus, adenovirus). Sputum and bronchoalveolar lavage (11) were processed for Gram and Ziehl–Neelsen stains and for cultures of bacterial, fungal and mycobacterial pathogens. Sputum samples were considered acceptable if there were more than 25 leukocytes and fewer than 10 squamous cells per low-power microscope field. Invasive samples were obtained if requested by the attending physician. Microorganism identification was consider positive as in previous publications. [9] Briefly, bacterial identification was achieved by means of the MALDI-TOF MS (Biomerieux, Marcy l’Etoile, France). Antimicrobial susceptibility was tested by the Kirby-Bauer disk diffusion technique on Muller-Hinton or sheep blood agar, depending on the microorganism growth requirements; E-test and in-house PCR were used to assess unexpected resistance patterns.

**Concept of Multidrug resistant pathogens (MDR)**  [10] MDR pathogens were classified according to European Centre of Diseases Prevention and Control: *P. aeruginosa* was considered MDR if non susceptible to at least 1 agent in 3 or more antimicrobial categories; MRSA was defined when *S aureus* was resistant to oxacillin corresponding to a minimum inhibitory concentration (MIC) of ≥4 mcg/mL. *Enterobacteriaceae* was defined as ESBL+ when they presented resistance to most β-lactam antibiotics, including penicillin, cephalosporins and aztreonam.

### Statistical analysis

#### Univariate analysis

Statistical analyses were performed using the SPSS software program 20.0. Qualitative variables were compared using the χ^2^ test. Quantitative variables were analyzed using the ANOVA test or the Kruskal-Wallis test. Values of p ≤0.05 were considered statistically significant. Length of stay was dichotomized as short (≤ 7 days) or long stay. FACED and BSI were dichotomized as severe (≥ 5 and ≥9 points respectively) and not severe.

#### Multivariate Analysis

Logistic regression analyses were performed to predict MDR pathogens as the dependent variable. Independent variables included were those found in the univariate analysis with *p* < 0.1. Variables that were highly correlated were excluded from the analysis. The subset of patients with non-MDR pathogens was used as the reference group. A second logistic regression analysis was also performed using as the reference group patients with non-MDR pathogens and patients without etiological diagnosis. The Hosmer and Lemeshow goodness-of-fit test was used to evaluate the adequacy of the models. [11] The areas under the receiver-operator characteristic (ROC) curves were also calculated.

## Results

### Patient characteristics

**Fig. 1:**
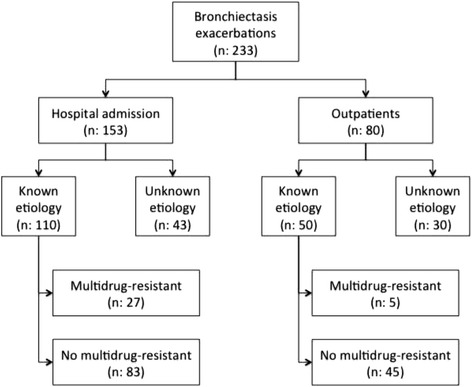
Flowchart

We recruited 233 patients with one exacerbation and microbial isolation was found in 159 of them (Fig. 1). Characteristics of patients, BE diagnosis, previous microorganisms isolations, number of exacerbations, number of prior antibiotic treatments, usual concomitant medications and severity scores are described in Table 1.

**Table 1:** Characteristics of patients according to isolation of multidrug-resistant microorganism in exacerbation

Characteristics		Multidrug-Resistant Microorganisms Isolated		
		No	Yes	*p* ^g^
Total No.		127 (79.9)	32 (20.1)	
Demographic data	Age	70 (62–77)	75 (68–79.5)	0.056
	>65 years	82 (64.6)	25 (78.1)	0.159
	Male	50 (39.4)	18 (56.2)	0.085
	Smoker or former smoker	58 (45.7)	18 (56.2)	0.284
	Alcohol abuse^a^	6 (4.7)	2 (6.2)	0.724
	Flu vaccine	89 (70.1)	18 (56.2)	0.136
Comorbid condition	Arterial hypertension	54 (42.5)	20 (62.5)	0.043
	Diabetes mellitus	20 (15.7)	7 (21.9)	0.409
	Myocardial infarction	11 (8.7)	1 (3.1)	0.289
	Congestive heart failure	14 (11)	8 (25)	0.041
	Cerebrovascular disease	3 (2.4)	1 (3.1)	0.805
	COPD^b^	32 (25.2)	14 (43.8)	0.039
	Asthma	12 (9.4)	3 (9.4)	0.990
	Previous pulmonary tuberculosis	11 (8.7)	4 (12.5)	0.507
	Renal disease	6 (4.7)	7 (21.9)	0.002
	Liver disease	8 (6.3)	2 (6.2)	0.984
	Age-adjusted Charlson >5	46 (36.2)	17 (53.1)	0.081
	Cystic bronchiectasis	7 (5.5)	4 (12.9)	0.147
	Chronic *Pseudomonas aeruginosa* infection	58 (45.7)	18 (56.2)	0.284
	Chronic infection by other microorganism	32 (25.2)	9 (29)	0.662
	Prior isolation of MDR^c^ microorganism	11 (8.7)	13 (40.6)	0.000
Treatment	Long-acting B-agonist	99 (78)	28 (87.5)	0.229
	Long-acting Anticholinergic	72 (56.7)	19 (59.4)	0.784
	Theophylline	5 (3.9)	3 (9.4)	0.208
	Inhaled corticosteroids	96 (75.6)	26 (81.2)	0.498
	Long term oral corticosteroids^d^	11 (8.7)	2 (6.2)	0.656
	Long term oral antibiotics	15 (11.8)	4 (12.5)	0.914
	Inhaled/Nebulized antibiotic	25 (19.7)	11 (34.4)	0.076
	Mucolytics	43 (33.9)	9 (28.1)	0.537
	Proton pump inhibitor	65 (51.2)	20 (62.5)	0.251
	Chronic oxygen therapy	14 (11)	9 (28.1)	0.014
	Regular chest physiotherapy	43 (33.9)	10 (31.2)	0.780
History of exacerbations	Hospitalization last year	62 (48.8)	26 (81.2)	0.001
	Previous history of pneumonia	67 (52.8)	14 (43.8)	0.362
	Exacerbation last year	98 (77.2)	28 (87.5)	0.198
	N° exacerbations last year	1 (1–2)	2 (1–3)	0.108
	Courses of antibiotic last year	1 (1–3)	2 (1–3)	0.366
Prognostic scales	Severe FACED^e^	16 (12.6)	9 (28.1)	0.031
	Severe BSI^f^	74 (58.3)	26 (81.2)	0.016

Data are presented as n (%) or median (interquartile range)

^a^Alcohol abuse: more than 80 g/day

^b^COPD: chronic obstructive pulmonary disease

^c^MDR: multidrug-resistant

^d^Long term oral steroids: less than 20 mg/day prednisone or equivalent

^e^FACED: FEV1, age, colonization, extension, dyspnea

^f^BSI: bronchiectasis severity index

^g^ *p* value: the χ^2^ test was performed for categorical data and the Mann-Whitney U test was performed for continuous data

### Microbiological results

The most frequent pathogens found during exacerbation are described in Table 2. MDR pathogens isolated during an exacerbation were found in 32 out of 241 microorganism, representing 13.2% of all microorganisms isolated: 16 (50%) MDR *Pseudomonas aeruginosa,* 6 (18.7%) methicillin-resistant *Staphylococcus aureus,* 2 (6.2%) extended-spectrum beta-lactamase-producing *Enterobacteriaceae* (*Proteus mirabilis* and *Serratia marcescens*) and 8 other bacteria (2 *Achromobacter xylosoxidans*, 2 *Stenotrophomonas maltophilia*, 1 *Brevundimonas diminuta*, 1 MDR *Escherichia coli* not ESBL*,* 1 *Haemophilus influenzae* ESBL and 1 MDR *Mycobacterium abscessus*).

**Table 2:** Microorganisms isolated in exacerbations

Microorganism Isolated	Total No. 241 (100)
*Pseudomonas aeruginosa*	51 (21.16)
^a^MDR *Pseudomonas aeruginosa*	16 (6.64)
Methicillin susceptible *Staphylococcus aureus*	11 (4.56)
Methicillin resistant *Staphylococcus aureus*	6 (2.49)
*Acinetobacter sp*	3 (1.24)
*Moraxella catarrhalis*	7 (2.9)
*Stenotrophomonas maltophilia*	4 (1.66)
*Enterobacteriaceae*	12 (4.98)
*Escherichia coli*	5 (2.07)
*Proteus spp*	3 (1.24)
*Klebsiella pneumonia*	3 (1.24)
*Serratia spp*	1 (0.41)
*Haemophilus influenzae*	27 (11.2)
*Streptococcus pneumoniae*	25 (10.37)
*Achromobacter xylosoxidans*	5 (2.07)
*Mycoplasma pneumoniae*	6 (2.49)
*Chlamydia pneumoniae*	1 (0.41)
*Atypical mycobacteria*	4 (1.66)
*Aspergillus spp*	12 (4.98)
*Candida spp*	15 (6.22)
Virus	25 (10.37)
Coronavirus	1 (0.41)
Metapneumovirus	4 (1.65)
Rhinovirus	10 (4.14)
Influenza A	3 (1.24)
Influenza B	2 (0.82)
Parainfluenza 3	2 (0.82)
Respiratory Syncytial virus	3 (1.24)
Others	11 (4.56)

^a^MDR: Multidrug-resistant

### Follow-up and outcome

Patients who required admission were more likely to grow MDR organisms than those who did not require admission (27/153 vs 5/80, p:0.016)(Fig. 1). Antibiotics initially prescribed for the exacerbation were changed in 37/159 patients, this occurred more frequently in those with MDR pathogens without reaching statistical significance (31.2% vs. 21.3%, p:0.23). In Table 3, there is depicted the outcome of exacerbations with regard to isolation MDR pathogens and patients without isolation are not included.

**Table 3:** Follow-up and outcome with regard to isolation multidrug-resistant pathogens or not in the exacerbation

Follow-up	No MDR^a^	MDR^a^	*p* ^*b*^
Complications	18 (14.9)	4 (12.5)	0.733
Change in the initial treatment	27 (21.3)	10 (31.2)	0.232
Adequate initial treatment	108 (85)	24 (75)	0.176
Length of stay	8 (6–11)	8 (6–14)	0.925
Exacerbation year	69 (56.1)	25 (62.5)	0.514

Data are presented as n (%) or median (interquartile range)

^a^MDR: Multidrug-resistant

^b^ *p* value: the χ^2^ test was performed for categorical data and the Mann-Whitney U test was performed for continuous data

### Risk factors for MDR pathogen exacerbations

#### Univariate results

Characteristics of patients, comorbid conditions, usual treatments, and scores regarding the presence or absence of MDR are shown in Table 1. Exacerbations were recorded and 153 of these patients were hospital admitted. MDR pathogens were more frequently encountered in patients with more chronic conditions and in those with higher FACED and BSI scores. No differences were found concerning usual prior treatments. Patients with prior hospitalization showed a significantly more frequent incidence of MDR.

#### Multivariate results

Three independent predictors to MDR exacerbations were identified. The area under the ROC curve for the model was 0.767 (95% CI, 0.669–0.865) (Table 4). In the second model using as a reference group patients with non-MDR pathogens and those without etiological diagnosis, these risk factors remained independently associated with MDR bacteria.

**Table 4:** Multivariate analysis to predict Multidrug-resistant pathogens

	Multidrug-Resistant Microorganisms		
	OR^a^	95% CI^b^	*p*
Age	1.03	0.97-1.09	0.393
Male	0.77	0.25–2.41	0.656
Arterial hypertension	0.83	0.27–2.62	0.756
Congestive heart failure	1.60	0.40–6.45	0.511
COPD	1.51	0.45–5.03	0.500
Renal disease	7.60	1.92–30.09	0.004
Age-adjusted Charlson >5	0.64	0.19–2.16	0.469
Chronic *Pseudomonas aeruginosa* infection	0.41	0.11–1.55	0.189
Prior multidrug-resistant microorganism isolation	5.58	2.02–15.46	0.001
Inhaled/Nebulized antibiotic	1.93	0.57–6.47	0.288
Chronic oxygen therapy	1.90	0.57–6.32	0.297
Hospitalization last year	3.88	1.37–11.02	0.011
Severe FACED	0.72	0.22–2.29	0.573
Severe BSI	1.58	0.42–5.95	0.501

^a^OR: Odds ratio

^b^CI: Confidence interval

### Probability of MDR and number of risk factors

**Fig. 2:**
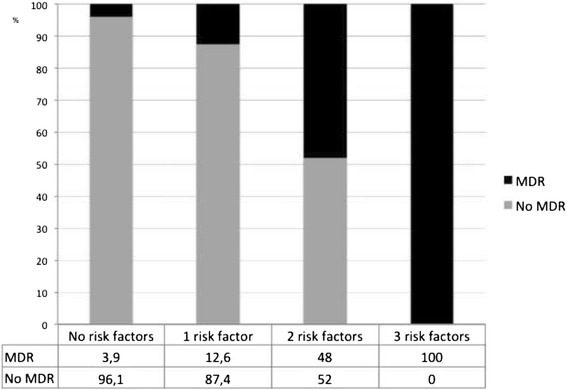
Number of independent MDR risk factors and percentage of MDR etiology

The presence of MDR in exacerbations with regard to the number of recognized risk factors found is shown in Fig. 2. No risk factors were identified in 102 patients and the probability of MDR in these patients was 3.9%. This probability increases to 12.6% when there is 1 risk factor and to 53.6% if 2 or more risk factors are present in total cohort.

## Discussion

The most notable findings of our study are the following: 1. MDR pathogens are frequently (20.1%) isolated in BE exacerbations, with a higher proportion among hospitalized patients (24.5%); 2. The principal MDR microorganisms were *Pseudomonas* (46%), MRSA and ESBL+ *Enterobacteriaceae;* 3. Independent MDR risk factors were prior MDR isolation, hospitalization in the previous year and chronic renal disease.

Due to the structural changes in permanently dilated airways, bronchiectasis courses with recurrent infections and exacerbations. Pathogens involved depend on several aspects: lung function, advanced phase of the disease and patient comorbidities. [1, 2, 12] However, little is known regarding frequency and factors associated with isolation MDR at exacerbations [13].

We found that in 20% of exacerbations MDR pathogens were isolated and the most frequent were *Pseudomonas,* MRSA and ESBL+ *Enterobacteriaceae*. We evaluated resistance using conventional methods usually performed in daily routine and we don’t perform automated methods or clonal analysis of resistance. [14] The percentage and spectrum of MDR is more similar to nosocomial than to community-acquired infections, in line with the current approach to those problematic pathogens based on host characteristics and prior treatments, [3, 13, 14] and slightly higher than that reported by McDonnell et al. [15] In fact, MDR exacerbations occurred in elderly patients with a higher proportion of comorbid conditions, indicating associations with more debilitating diseases, requiring more contacts with health resources. Our results showed that the use of prior inhaled antibiotics and long-term oxygen therapy was greater in patients with MDR pathogens. Interestingly, no differences were found with regard to the use of bronchodilators or inhaled corticosteroids. Metersky et al. [16] have reported that, in health-care associated pneumonia, inhaled corticosteroids were associated with *Pseudomonas* etiology, although they found no association with resistance.

The spectrum of microorganisms identified, [17] whether treated as outpatients or in hospital, was similar except for the fact that MDR was barely encountered in outpatients. [18] The fact that MDR exacerbations were more frequently admitted is clinically relevant because exacerbations that require hospitalization have been reported to be associated with an increase in 1-year mortality. [19]

In our study, we found three independent MDR risk factors: renal disease, prior MDR isolation and hospitalization in the previous year. Chronic renal disease is a recognized MDR risk factor, as reported in pneumonia studies. [3] Shindo et al., [20] identified 6 independent MDR risk factors, regardless of whether the patient has health-care associated or community-acquired pneumonia, suggesting that risk factors relied more on host factors than on the setting of infection. Prior hospitalization is a fairly widely recognized independent MDR risk factor and specifically for MRSA, [21] and for *Enterobacteriacea* mainly related to exposure to III/IV generation of cephalosporins or broad-spectrum penicillins. [22]

Prior MDR isolation was independently associated with a higher risk of MDR exacerbation. In our cohort, approximately 50% of patients had chronic *Pseudomonas* infection, [23] reflecting the most severe patients seen in a specific BE clinic. Prior MDR colonization is a recognized risk factor for MRSA [24, 25] and for *Pseudomonas* [26] in COPD patients. We found that 40% of patients with MDR exacerbations had prior isolation with the same microorganism.

The proportion of MDR exacerbations was higher among those patients with higher FACED and BSI scores, as expected in more advanced BE disease, with more proportion of exacerbations and hospitalizations**.** Almost 80% of MDR exacerbations occurred in patients with higher punctuations in prognostic scales such as FACED or BSI whereas MDR in mild scales were lower 6.2% and 40.6% respectively. However, after entering in the model other independent factors, these scales are not remaining independently associated with multi-drug resistance.

With regard antimicrobial susceptibility, MDR exacerbations received less appropriate treatment than non-MDR, thus also requiring more changes in antibiotic regimens although without statistical differences. In fact, the choice of initial treatment was microbiological suitable in 75% of cases, probably because physicians took into consideration prior MDR colonization, [27] a policy that is supported by our findings. Currently, factors considered in the antibiotic selection include extent of the disease, severity, local resistance patterns, and prior culture results. [28] A practical conclusion is that extended-spectrum antibiotics against MDR could be withdrawn in patients with no risk factors and indicated if 2 or more risk factors are present. Where 1 risk factor is present, an extended-spectrum antibiotic may be indicated until MDR pathogens have been ruled out; a microbiological work-up should therefore be implemented. [29] Nevertheless, these recommendations need to be validated in different populations or BE subsets [30] and knowledge of local resistance rates and colonization rates should be considered. This policy may contribute to containing broad-spectrum coverage for MDR in unnecessary episodes and this strategy may contribute to curbing the future emergence of resistant microorganisms in this population.

Patients with MDR exacerbations required more hospitalizations and greater use of antibiotics although without longer hospital stay. In general, MDR infections have been associated with a higher number of days of hospitalization, [31] with higher antibiotic requirements, more hospitalization, [32] more use of health resources, with the attendant higher costs, and may eventually have a negative impact on prognosis. [33] Nevertheless, we consider that one-year follow-up could be insufficient for evaluating the potential clinical impact of MDR exacerbations and probably for that aim more subsequent exacerbations should be assessed.

### Limitations

Pathogen identification relied mainly on conventional microbiological tests and invasive respiratory samples were only indicated if required by the attending physician; this is a real clinical scenario common in clinical hospital settings. No quantitative bacteriology measuring with colony counts was quantified in sputum. Due to the number of patients in the cohort, a secondary analysis to separate specific risk factors for each microorganism was not undertaken. Mild episodes of exacerbations treated in primary care and not evaluated in our specific clinics were not included.

### Strengths

This is the first study aimed at identifying risk factors for MDR exacerbations with potential impact on clinical decisions for antibiotic choice. At present, BTS guidelines [28] suggest combination therapy rather than single-drug antibiotic therapy if a resistant strain of *P aeruginosa* is isolated. Our findings could be useful for avoiding unnecessary broad-spectrum antibiotics in patients without MDR risk factors.

## Conclusions

Our findings have identified three independent risk factors - hospitalization in the previous year, chronic renal disease, and prior multidrug-resistant isolation- for identification multidrug-resistant pathogens in BE exacerbations. This information may be useful for clinicians in guiding initial antibiotic therapy in exacerbations of BE. A further validation in different BE cohorts including distinct phenotypes and larger follow-up periods should be performed. MDR risk prediction in BE exacerbations is a new field that requires validation for clinical decision-making in selecting initial appropriate antibiotics and for safely avoiding anti-MDR coverage.

## Abbreviations

AIDS: Acquired immunodeficiency syndrome
BE: Non-cystic fibrosis bronchiectasis
BSI: Bronchiectasis severity index
BTS: British Thoracic Society
CF: Cystic fibrosis
COPD: Chronic obstructive pulmonary disease
ESBL: Extended-spectrum betalactamase
FACED: F (forced expiratory volume in 1 s [FEV1]); A (age; C: chronic colonization by Pseudomonas aeruginosa [PA]); E (radiological extension [number of pulmonary lobes affected]); and D (dyspnea)
HIV: Human immunodeficiency virus
MDR: Multidrug-resistant
MIC: Minimum inhibitory concentration
MRSA: *Methicillin-resistant Staphylococcus aureus*
PA: *Pseudomonas aeruginosa*
ROC: Receiver-operator characteristic
